# Synthesis of Biocompatible and Biodegradable Polyamidoamines Microgels via a Simple and Reliable Statistical Approach

**DOI:** 10.3390/ma15207280

**Published:** 2022-10-18

**Authors:** Nicolò Mauro, Gaetano Giammona, Elisabetta Ranucci, Paolo Ferruti

**Affiliations:** 1 Laboratory of Biocompatible Polymers, Department of “Scienze e Tecnologie Biologiche, Chimiche e Farmaceutiche” (STEBICEF), University of Palermo, 90123 Palermo, Italy; 2 Dipartimento di Chimica, Università degli Studi di Milano, 20133 Milano, Italy

**Keywords:** polyamidoamines, microgels, drug delivery, polyaddition, biodegradable polymers

## Abstract

Polyamidoamines (PAAs) are biocompatible and biodegradable polymers with a huge potential as biomaterials for pharmaceutical applications. They are obtained by the step-wise aza-Michael polyaddition of bifunctional or multifunctional amines with bisacrylamides in water. To the best of our knowledge, no synthetic protocols leading to hyperbranched PAAs as well as PAA microgels have been published so far. To fill this gap, a statistical approach was established in this work to fine-tune the aza-Michael polyaddition stoichiometry when a multifunctional co-monomer (b_f_) is added to a mixture of bifunctional monomers with complementary functions (a_2_ + b_2_), possibly even in presence of a monofunctional co-monomer (b_1_), for obtaining either microgels or hyperbranched polymers by a one-pot reaction. For this purpose, two new equations, obtained by reworking the classic Flory–Stockmayer equations, were successfully applied to the synthesis of different model systems, obtaining biocompatible microgels with tunable size distribution (200–500 nm) and properly designed end-chains in a simple and straightforward way. The same mathematical approach allowed us to empirically evaluate the actual number of active reactive functions of the co-monomers. A number of selected systems, being evaluated for their cytotoxicity in vitro, proved highly cytocompatible and, therefore, endowed with great potential for pharmaceutical and medical applications.

## 1. Introduction

**Scheme 1:**
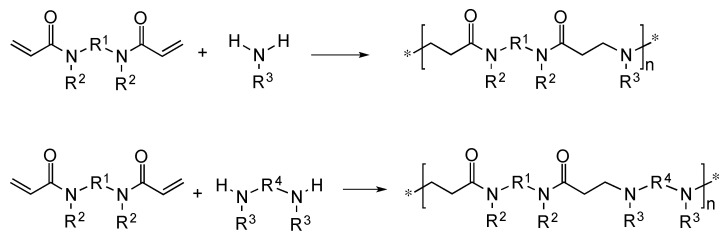
Synthesis of PAAs.

Polyamidoamines (PAAs) are a class of biodegradable and biocompatible polymers obtained by the stepwise aza-Michael polyaddition of *prim*-monoamines or *sec*-diamines with bisacrylamides (Scheme 1) [1]. They are promising polymers, useful to prepare biomaterials for many biomedical and pharmaceutical applications since, apart from their high biocompatibility and tunable biodegradability, they are extremely versatile [2]. In fact, countless PAAs with tunable biological and physicochemical properties can be in principle designed by changing the combinations of starting monomers and stoichiometric conditions [3,4,5,6].

Incidentally, PAAs are not the only polymers obtained by the aza-Michael polyadditions of amines with acrylamides. In fact, the self-polycondensation of 2-aminoethylacrylamide, an “ab_2_” type monomer, that is, a monomer carrying complementary “a” and “b” functions in a 1:2 ratio, taking place by alkalinization of 2-aminoethylacrylamide hydrochloride, gives rise to polyamidoamine dendrimers named PAMAMs [7]. PAMAMs should not be confused with PAAs, since their molecular architecture is different. In PAMAMs, each amide group (the “a” function) is preceded and followed by an amine group (the “b” function), giving rise to the (ab)_n_ sequence. Conversely, in the PAA backbone, the sequence of the amide and amine groups is either (aab)_n_ or (aabb)_n_, depending on whether *prim*-amines or bis-*sec*-amines were involved in their synthetic protocols [1].

PAA-based nano-sized systems have been widely studied for applications in the biological and biomedical fields. For instance, PAAs have been found to be able to complex and transport genes, siRNA, and proteins into different cytotypes enabling the specific release of many bioactive compounds at the subcellular level [8,9]. Furthermore, PAAs containing hydrophobic moieties as side substituents have been shown to form nanoparticles with a hydrophobic core capable of solubilizing and releasing various hydrophobic drugs [10]. Until now, mainly linear PAAs have been considered for these purposes, although highly branched macromolecules and microgels have proved to be more effective due to the smaller size, more compact shape, and high concentration of terminal groups on their surface. In addition, branched polymers usually exhibit much lower solution viscosity than the corresponding linear polymers having the same molar mass, a characteristic that may facilitate technological, manufacturing, and medical processes, including administration. To the best of our knowledge, while much information is available on PAMAMs [7], no clear reports have been published so far on synthetic protocols to obtain PAA microgels or hyperbranched PAAs.

**Figure 1:**
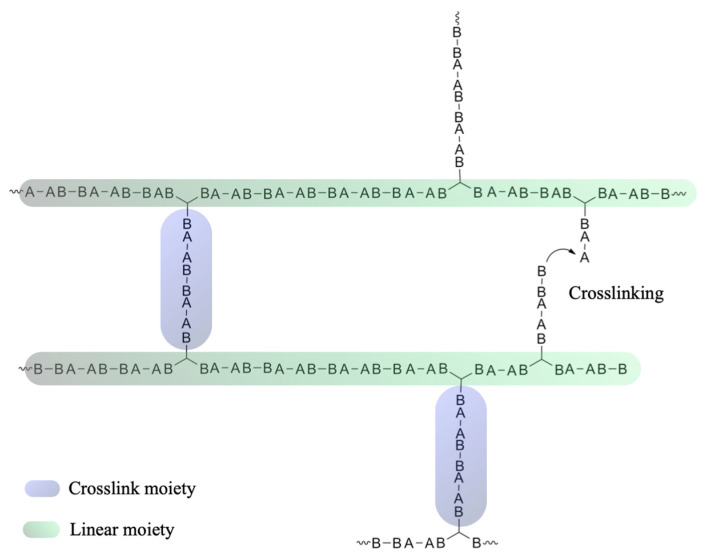
Structure of a cross-linked macromolecule obtained from the polyaddition of divalent monomers with “a” and “b” functions, a_2_ and b_2_, and a trivalent monomer bearing “b” functions b_3_.

Hydrophilic cross-linked PAAs in the form of soft hydrogels have been studied as scaffolds for tissue engineering [11] as well as heavy metal ions absorbing resins [12]. The easiest way for preparing cross-linked PAAs is to partially or totally substitute amines in their polymerization recipes bearing three or more reactive hydrogen atoms for the primary monoamines or the secondary bis-amines normally employed as monomers (Figure 1) [1]. In principle, stable PAA microgels for pharmaceutical applications could be prepared by finely milling bulky hydrogels. However, this process is expensive and leads to polydisperse microgels with indefinite morphology, thus limiting their practical application. The preparation of nanogels for drug administration from self-assembled reversible zwitterionic PAAs has also been described [13].

In this work, theoretical considerations have been used to predict if, in a given step-wise polymerization system, gelation occurs as a function of the feed and the advancement degree of the polymerization reaction. It was found that a reworking of the classic Flory–Stockmayer equations [14,15,16] allowed for the prediction of whether, under the condition of complete conversion, soluble hyperbranched PAAs or PAA microgels with potential for pharmaceutical application could be obtained by the aza-Michael polyaddition of bisacrylamides with multifunctional amines by adjusting the molar ratio between multifunctional and bifunctional co-monomers in the feed. The validity of the adopted mathematical model was confirmed by a set of experiments performed with different model systems. The aim of this paper is to report on the results obtained.

## 2. Materials and Methods

Piperazine (99.5%), lithium hydroxide (99.5%), ethylene diamine (EDA) (99.8%), cyclam (Cy) (99.5%), morpholine (99%), carbon tapes, hydrochlric acid (37 %), sodium chloride (99.5%), and tris buffer were purchased by Sigma Aldrich (Milan, Italy) and used as received. 2,2′-bisacrylamidoacetic acid (BAC) (98.4%) was obtained as previously described [17]. Cell Titer 96 Aqueous One Solution Cell Proliferation assay (MTS solution) were purchased from Promega (Milan, Italy).

### 2.1. General Polymerization Procedure

BAC (555.8 mg, 2.6250 mmol, purity 93.6%) was dissolved in deoxygenated doubly distilled water (1.00 mL) under nitrogen atmosphere in the presence of lithium hydroxide (63.5 mg, 2.6250 mmol). The amines (EDA, 22.8 mg, 0.3750 mmol; and P, 254.2 mg, 3.0000 mmol) were then added at once all together and the mixture gently stirred until homogeneous and maintained at 20 °C under nitrogen for 7 days with stirring. After this time, the reaction mixture was acidified to pH 4 (1 M HCl). The product, if soluble, was purified by dissolving in water and ultrafiltering through a membrane with nominal cut-off 1000 (Merk Millipore, Milan, Italy). It was finally recovered by freeze-drying (Labconco, Milan, Italy) and the yield determined. Characterizations were performed on the final product. In the case of gel formation, the gel time was estimated, and the polymerization was then allowed to proceed as described. The final product, after acidification, was thoroughly extracted with water, dried, and the yield determined. The soluble extracts, if any, were not further characterized. The exact amounts of all reagents for each preparation are reported in Table 1 and Table 2.

### 2.2. Size Exclusion Chromatography (SEC)

SEC traces were obtained using a Knauer Pump 1000 (Berlin, Germany) equipped with a Knauer Autosampler 3800, TKSgel G4000 PW, and G3000 PW TosoHaas columns connected in series, using a light scattering (LS)/viscosimeter Viscotek 270 Dual Detector (Cambridge, UK), coupled to a refractive index detector (Waters model 2410 Milford, USA). The mobile phase was a 0.1 M Tris buffer (pH 8.0 ± 0.05 with 0.2 M sodium chloride. The sample concentration was 2% (*w*/*v*) and the flow rate was 1 mL min^−1^. Triple detector SEC allowed determining, besides the molecular weight, the long chain branching (LCB) frequency of star-like PAAs by means of the dedicated Viskotech software (Omnisec, 2001, Cambridge, UK), which compared their Mark–Houwink plots with those of samples of their linear counterparts of approximately the same molecular weight and used common Zimm–Stockmayer equation and methods.

### 2.3. Dynamic Light Scattering (DLS) Analysis

Dynamic light scattering analyses were performed on microgels dispersed in ultrapure water (1 mL, 0.1 mg mL^−1^) at 25 °C by a Malvern Zetasizer NanoZS instrument (Rome, Italy) equipped with a 632 nm laser with a scattering angle of 173°, and the Dispersion Technology Software 7.02 software (Malvern Panalytical Ltd., Almelo, The Netherlands). Zeta-potential measurements were performed by aqueous electrophoresis measurements, recorded at 25 °C using the same apparatus for the DLS measurement. The Zeta-potential values (mV) were calculated from electrophoretic mobility using the Smoluchowski relationship. All analyses were performed in triplicate, and data are reported as average values.

### 2.4. Scanning Electron Microscopy (SEM)

Scanning electron microscopy was performed on non-porous carbon tapes using a FEI Versa 3D (FEI, Hillsboro, OR, USA) microscope operating at 0.5–30 kV. Briefly, samples were dispersed in ultrapure water (0.1 μg mL^−1^) and deposited on carbon tape surface (20 μL,) to be dried under vacuum (10 mbar) over night. After that, stubs were retrieved, sputtered with gold nanoparticles of about 5 nm, and observed under high vacuum by using a secondary electron detector.

### 2.5. Cell Culture and Cell Viability Assay

The human dermal fibroblasts (HDFa) cell line was purchased from Sigma Aldrich (Milan, Italy) and cultured in Dulbecco’s Minimum Essential Medium (DMEM) supplemented with 10% fetal bovine serum (FBS, Euroclone, Milan, Italy), 1% of penicillin/streptomycin (10,000 U mL^−1^ and 10 mg mL^−1^ respectively, Euroclone, Milan, Italy), and 1% of L-glutamine (Euroclone, Milan, Italy), at 37 °C in 5% CO_2_ humidified atmosphere.

For the cell viability assay, HDFa were seeded in a 96-multiwell plate with a density of 2 × 10^4^ cells/well (200 µL/well) and incubated at 37 °C and 5 % of CO_2_ (Air Liquide, Milan, Italy). After 24 h, the culture medium of each well was replaced with a dispersion of microgels (150 µL/well) at different concentrations (0.01–1 mg mL^−1^), and cells were incubated for 48 h. Then, each well was washed up with DPBS (200 µL × 3), an MTS solution in fresh culture medium (20 µL of MTS and 100 µL of DMEM) was added to each well, and cells were incubated for an additional 3 h at 37 °C and 5 % of CO_2_. Cell viability was obtained by measuring the absorbance at λ492 using a microplate reader (Multiskan, Thermo Fisher Scientific, Waltham, MA, USA) and considering untreated cells as 100% of cell viability. All biological experiments were performed in triplicate and results are reported as mean values.

## 3. Results and Discussion

### 3.1. Theoretical Considerations

According to the classic Flory–Stockmayer theory [14,15,16], the cross-linking and gelation of step-growth polymers can be predicted by Equation (1):

(1) pc=1{r×[1+ρ×(f−2)]}12

where *p_c_* is the so-called critical advancement degree, above which the reacting system loses its mobility giving rise to a gel; *r* is the stoichiometric ratio in the feed between the complementary “a” and “b” reactive functions involved in the polymerization reaction; *ρ* is the fraction of functions belonging to the polyfunctional monomer over the total amount of same functions in the feed; and *f is* the number of functions of the polyfunctional monomer.

Since the advancement degree of any reaction cannot obviously be higher than 1, it has been hypothesized here that setting *p_c_* = 1 in Equation (1) and then solving for *r*, it was possible to obtain the critical molar ratio in the feed, *r_c_*, above which gelation takes place at 100% conversion (*p* = 1) and below which the system cannot cross-link even at *p* = 1, but can only form soluble hyperbranched products. The *r_c_* value, obtained by reworking Equation (1) as above stated, is given by Equation (2):

(2) $$r_{c}=\frac{1}{1+\rho \times \left(f-2\right)}$$

Equations (1) and (2) apply to systems of the type a_2_ + b_2_ + b_f_, where *f* > 2, and to monomers endowed with similar reactivity. They cannot be applied to systems where monofunctional co-monomers of the type a_1_ or b_1_, which obviously act as chain terminators, are present in the feed. Monofunctional co-monomers are indeed often employed in polyaddition to tune the molecular weight of polymers by establishing the terminals of the resulting macromolecules. In the presence of monofunctional co-monomers, the critical advancement degree of the system can be predicted by Equation (3), statistically derived from the Flory equation [18,19]:

(3) pc=1[r×(fw,a−1)×(fw,b−1)]12

where $f_{w,a}$ and $f_{w,b}$, the weight average monomer functionalities, including the monofunctional ones, are given by Equations (4) and (5):

(4) $$f_{w,a}=\frac{\sum_{j=1}^{n}f_{a,j}^{2}\times N_{a,j}}{\sum_{j=1}^{n}f_{a,j}\times N_{a,j}}$$

(5) $$f_{w,b}=\frac{\sum_{j=1}^{n}f_{b,j}^{2}\times N_{b,j}}{\sum_{j=1}^{n}f_{b,j}\times N_{b,j}}$$

where $f_{a,j}\;$and $f_{b,j}\;$represent the functionalities of each monomer of *a* or *b* type, respectively, and $N_{a,j}$ and $N_{b,j}$ are the corresponding mole number in the system. Similar to Equation (1), Equation (3) can be reworked to define a new critical molar ratio in feed, *r_mc_*, above which the system can reach the gel point and below which it can only form soluble hyperbranched polymers, valid when monofunctional co-monomers are present. In fact, setting *p_c_* = 1 in Equation (3), and solving for *r*, the *r_mc_* value can be obtained, according to Equation (6):

(6) $$r_{mc}=\frac{1}{\left(f_{w,a}-1\right)\times \left(f_{w,b}-1\right)}$$

It should be emphasized that, although the classic method based on *p* control (see Equation (1)) has so far received much attention [20], in practical terms it is of rather difficult application due to the need of perfect knowledge and fine control of the polymerization kinetics during all reaction stages. Conversely, the new method here reported (see Equations (2) and (6)) is solely based on the adjustment of the molar ratio between the reactive functions of monofunctional and polyfunctional co-monomers, and the completion of the polymerization reaction (*p* *≈* 1). Therefore, it can be easily applied to co-monomers of a wide range of reactivity. On the top of that, this model can be used to design the synthesis of soluble hyperbranched polymers (*r* < *r*_c_) or gels (*r* > *r*_c_).

### 3.2. Systems Adopted and Rationale of the Approach

**Scheme 2:**
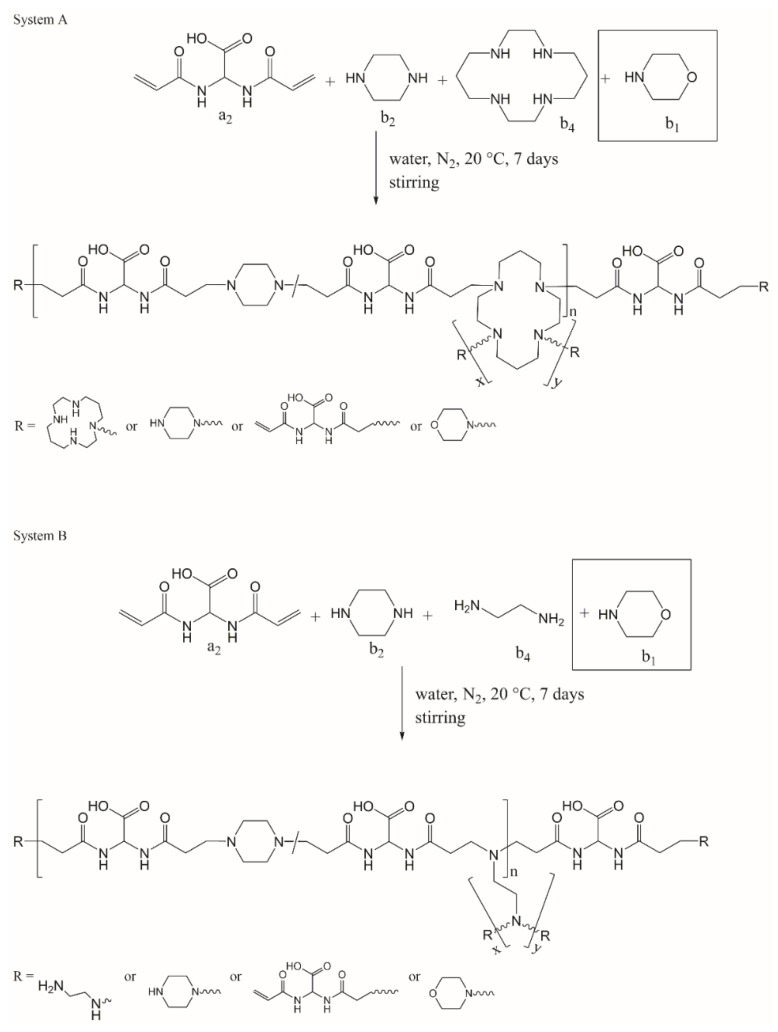
Synthetic pathway adopted for the System A- and System B-based PAAs: morpholine was employed as end-chain group only for some reaction where the monomer b_1_ was considered.

The rationale of the approach adopted is to evaluate whether by appropriately adjusting the overall stoichiometry of a_2_ + b_2_+ b_f_ as well as of a_2_ + b_2_+ b_f_ + b_1_ type systems it is possible to obtain in a predictable way PAA microgels or soluble hyperbranched PAAs with controlled macromolecular sizes and architectures. As regards the mathematical models, the above reported Flory–Stockmayer equations are valid only if all functions of the same type present in the monomers are equally reactive. In the present work, it has been hypothesized that, for the aza-Michael polyaddition leading to PAAs, this condition was satisfied by employing symmetrical bisacrylamides, -bis-*sec*-amines and -macrocyclic polyamines with a relatively long distance between the reactive functions, together with a highly reactive monoamine. The requirement of the equal reactivity of the functional groups was met by a first model system (System A) consisting of 2,2′-bis(acrylamido)acetic acid as bisacrylamide (a_2_), piperazine as a difunctional amine (b_2_), and 1,4,8,12-tetraazacyclotetradecane (cyclam), a cyclic tetrafunctional amine (b_4_) whose amine N-H are well-isolated from each other and, therefore, were expected to be equally reactive in polyaddition (Scheme 2). A total of seven polyaddition experiments were performed (see Table 1), out of which four using the amine N-H (1.1–1.3 and 1.5) and two the acrylamide double bonds (1.4 and 1.6) as the excess functions. In one case (1.7), also morpholine (b_1_) was employed as a monofunctional terminating monomer. Only one value of *r* and one of *r_m_*_c_ have been chosen.

In a second model system (System B), a different tetrafunctional amine (b_4_), that is, 1,2-aminoethane (or ethylenediamine, EDA) was used as cross-linking agent in place of cyclam (Scheme 2). It should be observed that, due to steric hindrance introduced by the aza-Michael addition, the four amine N-H functions of EDA were expected to have different reactivities after each addition step. Notwithstanding, the different reactivities of the four amine N-H of EDA had previously been shown not to be as high as to hinder its use as cross-linking agent. In fact, EDA had been extensively used for this purpose with good results over the past decade [1,21]. Therefore, EDA was tested in the following calculations considering it both as a tetrafunctional monomer (*f* = 4) and as a trifunctional monomer (*f* = 3) (see Section 3.3). The aim was to test the suitability of the above mathematical model to identify the number of reactive functions that actually participate in the aza-Michael polyaddition reaction. To this purpose, a total of 13 polyaddition experiments were performed (reported both in Table 2 and Table 3), out of which 8 using the amine N-H (in Table 2: 2.1–2.4 and 2.8–2.11) and 2 the acrylamide double bond (in Table 2: 2.5–2.7 and 2.12) as the excess functions. In two cases (in Table 2: 2.8 and 2.13), also morpholine (b_1_) was employed as a monofunctional terminating monomer. Two different values of *r* and two of *r_mc_* have been chosen.

In all cases considered, the reaction conditions were set so as to reach *p* ≈ 1.

### 3.3. Compliance with the Flory–Stockmayer Model

Results obtained for System A, in which cyclam was used as polyfunctional co-monomer (*f* = 4), are reported in Table 1. Interestingly, in line with the mathematical models adopted (Equations (2) and (6)), polyaddition experiments using both acrylamide double bonds and aminic N-H as excess functions gave invariably microgels when *r* > *r_c_*, whereas soluble hyperbranched polymers were obtained when *r* < *r_c_* (or *r* < *r_mc_*), showing no evidence of the presence of microgels or nanogels as revealed by dynamic light scattering analysis. These data, besides further confirming the correctness of Equations (2) and (6), prove that cyclam behaves as a tetrafunctional monomer with homogeneous reactivity of the four amine N-H.

As regards System B, calculations have been performed considering EDA both as a tetrafunctional monomer (*f* = 4) and as a trifunctional monomer (*f* = 3). Results are shown in Table 2 and Table 3, respectively.

Because EDA is a polyfunctional monomer with different reactivities of the aminic N-H in the aza-Michael addition reaction, conflicting results were obtained for the polyaddition experiments performed with excess aminic N-H (Table 2, 2.1–2.4, 2.8–2.11, and 2.13) when an *f* = 4 value was assumed. In this instance, when *r* > *r_c_* (or *r_mc_*) the polyaddition reaction led to soluble hyperbranched PAAs, with the only exceptions being experiments 2.3, 2.5, and 2.6. Conversely, the polyadditions experiments carried out with excess acrylamide functions gave invariably microgels, in full agreement with the interpretation of the classic Flory–Stockmayer theory expressed in Equations (2) and (6). It was postulated that the excess acrylamide functions allowed the addition of all four amine N-H, circumventing the effect of their different reactivities.

Conversely, when *r*, *r_c_*, and *r_mc_* were calculated by hypothesizing that EDA reacts as a trifunctional monomer, that is, when an *f* = 3 value was assumed, all results correlated well with the theory both when experiments were performed with excess acrylamide functions and with excess amine N-H (Table 3, 3.1–3.4, 3.8–3.11, and 3.13).

In conclusion, the above results confirm that three of the four aminic N-H are significantly more reactive than the fourth one, due to the steric hindrance introduced by the aza-Michael additions. The first two amine N-H belong in fact to primary amines and can reasonably be expected to be highly reactive, while the remaining two, belonging to secondary amines, should be less reactive, albeit with some qualifications. In fact, when three out of four amine N-Hs have reacted, the fourth is even more hindered and, therefore, reacts even more slowly.

According to theory, hyperbranched PAAs obtained under non-gelling conditions should have a modest average polymerization degree (DP_n_ ≈ 4), coupled with high polydispersity (PD ≈ 10) [20]. We did not try to study in detail the compliance of our systems with these theoretical aspects. Instead, we aimed at establishing a synthetic protocol for preparing microgels of hyperbranched PAAs with suitable average molecular weight and narrow-to-moderate molecular weight distribution. To this purpose, we fractionated hyperbranched PAAs by ultrafiltration using a membrane with cut-off 1000, and analyzed only fractions retained and retrieved after freeze-drying. The molecular weights of the resultant PAAs were obtained by SEC-LALS/RALS and reported in Table 1, Table 2 and Table 3. The reaction yield was roughly 45–55%. It may be observed that the final products were obtained in relatively low yields and with low molecular weights (about a few thousands), that is significantly lower than that usually obtained for linear PAAs. However, this method allows synthesizing multifunctional PAAs bearing multiple end-chains of the same kind, either amine or acrylamides, according to the excess of reagent employed in the starting polymerization recipe. This opens the way to further functionalization useful for the synthesis PAAs with tailor-made properties.

On the whole, considering the nature of the polyfunctional polymer and the stoichiometric conditions established at the starting point, one can design microgels with various size distribution and properties or hyperbranched PAAs with tunable macromolecular architecture. This is a powerful method to obtain PAAs helpful in many medical applications such as drug delivery and tissue engineering.

### 3.4. Physicochemical Characterization of Selected Microgels

The synthetic protocols adopted using both systems allow foreseeing the formation of PAA microgels by tuning starting monomers and stoichiometric conditions. These would be useful as drug delivery systems, and in situ gelling biomaterials for tissue engineering applications. As shown in Table 4 the size distribution of microgels of both series is within the range 311–528 nm. Dynamic light scattering provides information on the hydrodynamic diameter in aqueous environment taking into account the hydration sphere of the polymer. This encloses the network of microgels and bonded water and gives a reliable size distribution under the physiological environment. It is interesting to notice that the microgels obtained have a suitable diameter to enter cells and tissues, serving as potential drug delivery systems. In addition, they can also serve as pulmonary delivery systems, having dimensions (100–500 nm) suitable to reach bronchioles [22,23].

Moreover, it seems that the size distribution of microgels of the A series varies by controlling the *r-r_c_* value of the starting reaction conditions. In particular, the size distribution increases by increasing *r-r_c_* values, passing from 398 nm to 528 nm for a *r-r_c_* of 0.016 (2.6) and *r-r_c_* of 0.241 (2.12), respectively. A similar trend is observed for microgels of the B series. As a rule, polydispersity did not follow a trivial trend and, as reported in Table 4, is within the range 0.19–0.29, implying the formation of homogeneous microgels.

DLS measurements also provide detailed information of the Zeta-potential of microgels, resembling the typical behavior of colloids at the cell-biomaterial interface. It is well-known that cationic colloids can strongly interact with cells, owing to inherently negative Nernst membrane potential of the latter. Here it has been shown that, as expected, the microgels obtained have a positive Zeta-potential, which guarantees stability in water and excellent interactions with cells both in vitro and in vivo.

**Figure 2:**
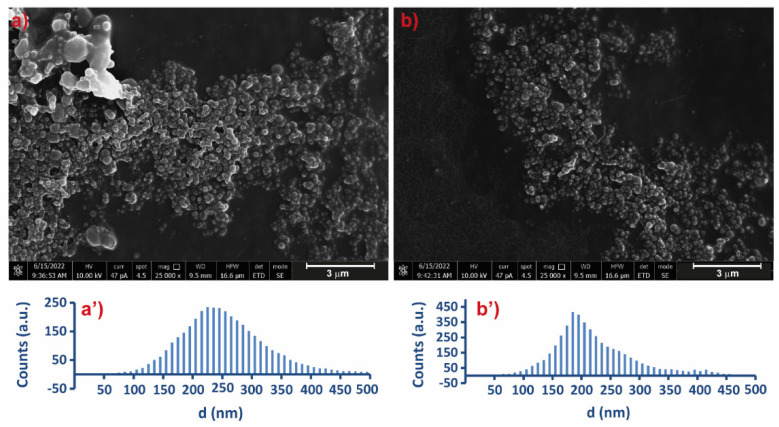
Scanning electron microscopy (SEM) of selected microgels of the B (2.3, (**a**)) and A (1.3, (**b**)) series: the size distribution of dried samples is reported on the bottom (**a’**,**b’**).

The size distribution and morphology of two selected microgels (one per each series) was studied by scanning electron microscopy (SEM) analysis (Figure 2a,b). Surprisingly, both microgels display quasi-spherical morphology and, according to DLS data, a narrow size distribution. Obviously, because of the removal of the hydration shell, the size distribution of microgels after freeze-drying appears smaller than that observed by DLS analysis (about 248 nm VS 481 and 197 nm VS 356 nm for the 1.3 and 2.3, respectively).

The observed morphology can be due to dilution of the starting reaction which facilitates collapsed but well-connected microgels instead of bulk hydrogels. Spherical microgels are suitable vehicles for pulmonary drug delivery applications, since the aerodynamic behavior of spherical particles can be easily described [24,25].

### 3.5. Cytocompatibility of the Selected Microgels

PAA hydrogels have been previously prepared and characterized for many biomedical applications, namely as scaffolds for bone and nerve regeneration [1,2,3,4,11]. However, there are no available data on cyclam-based microgels and comparison with the most common EDA-based ones. Here, two main microgel samples were selected, one of the A series and one of the B series, in order to test their cytocompatibility, and thus their potential as biomaterials for pharmaceutical applications. The cell viability of human dermal fibroblasts (HDFa) following exposure to increasing amount of microgels was assessed. Results were compared to untreated controls and cell viability was calculated considering controls as 100% cell viability.

**Figure 3:**
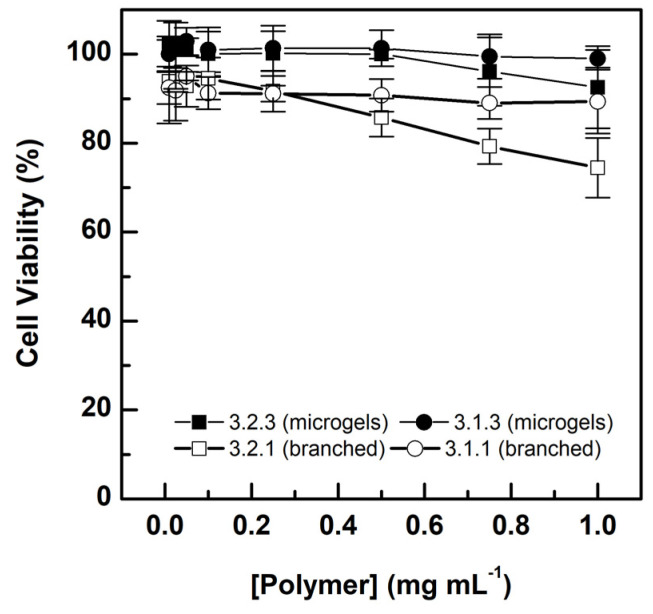
Cell viability of human dermal fibroblasts (HDFa) treated with some selected microgels (solid symbols) and hyperbranced PAAs (open symbols). EDA series (circle) and cyclam series (square).

Cell viability of HDFa treated with both microgel types was always higher than 89% within the entire concentration range considered (Figure 3, solid symbols), that is, above the threshold of 70% [26]. This implies that both microgel types are cytocompatible and, therefore, have a great potential in view of pharmaceutical and medical applications. However, it may be observed that microgels obtained using EDA as multifunctional monomer show a cell viability higher than those synthesized using cyclam (89% vs. 99 %, respectively). This can be due to the higher *pKa* values of cyclam (about 11.5), which usually implies higher toxicity [27]. This trend is much more evident in soluble hyperbranched PAAs, where the EDA-containing PAA appears much more cytocompatible when compared with the cyclam series (cell viability at the higher concentration tested 88% vs. 76%) (Figure 3, open symbols). On the whole, the synthetic strategy adopted allows for developing biocompatible PAAs microgels and polymers with a well-established tridimensional network and tunable physicochemical and biological properties. This will pave the way to a fruitful development of new engineered PAA microgels for many different purposes.

## 4. Conclusions

In this work, a reliable procedure was devised for obtaining at will either cross-linked PAA gels or hyperbranched soluble PAA gels with controlled chain ends from mixtures of monomers that include multifunctional monomers, bifunctional monomers with complementary functions and, in special cases, monofunctional co-monomers. This procedure is of general application and is based on the reworking of the classic Flory–Stockmayer equation relating the critical advancement degree of the polymerization reaction (*p_c_*) with the molar ratio *r* of the complementary reactive functions. New equations were obtained in which an additional parameter (*r_c_*) was defined as the critical compositional molar ratio, that is, the ratio of the reactive functions above which the system reaches gelation and below which it only gives soluble hyperbranched polymers. The devised procedure is valid under the conditions that complete conversion (*p* ≈ 1) is achieved and, moreover, is based on a simple one-pot process. To the best of our knowledge, no similar procedures have been published so far.

It may be noticed that the application of the new equations is based solely on the identification of the *r_c_* parameter. They were successfully applied to the synthesis of different model systems of increasing complexity, including not only multifunctional monomers with approximately equal reactivity, but also multifunctional monomers with unequal reactivities of the different functions, as well as monofunctional co-monomers acting as chain terminators. Interestingly, the new equations and related procedures allow for the identification of the actual number of equally reactive functions in the multifunctional co-monomer, thus allowing prediction of the macromolecular architecture of the final products even in this case.

PAA microgels with tunable size and homogeneous size distribution (200–500 nm) were produced under gelation conditions by simply mixing the reaction vessel. Selected products, once in vitro evaluated for their cytotoxicity, proved highly cytocompatible. All the above results point to the conclusion that the new PAA products are endowed with remarkable potential for pharmaceutical and medical applications.

## Figures and Tables

**Table 1:** Results obtained for System A, based on 2,2′-bis(acrylamido)acetic acid (BAC, a_2_), piperazine (P, b_2_), cyclam (Cy, b_4_), and morpholine (M, b_1_).

N°	BAC (mmol)	Cy (mmol)	P (mmol)	M (mmol)	Excess Reactive Function ^(a)^	*r_c_* ^(b)^	*r* ^(c)^	*r-r_c_*	${\bar{M}}_{n}$ (10^−3^)	${\bar{M}}_{w}$ (10^−3^)
1.1	2.6250	0.3750	3.0000	-	N-H	0.714	0.700	−0.014	4.7	6.1
1.2	2.4975	0.3750	3.0000	-	N-H	0.714	0.667	−0.047	3.9	5.7
1.3	3.1875	0.3750	3.0000	-	N-H	0.714	0.850	+0.136	^(d)^	^(d)^
1.4	1.5630	0.1250	1.0000	-	=	0.714	0.800	+0.086	^(d)^	^(d)^
1.5	1.0004	0.1250	1.0000	-	N-H	0.714	0.800	+0.086	^(d)^	^(d)^
1.6	1.7863	0.1250	1.0000	-	=	0.714	0.709	−0.005	3.7	9.1
1.7	3.9280	0.4550	3.0000	2.0000	N-H	0.857 ^(e)^	0.800	−0.057	3.4	4.6

^(a)^ N-H: amine function; = acrylamide functions. ^(b)^ Critical compositional ratio obtained from Equation (4) assuming *f* = 4. ^(c)^ Compositional ratio adopted. ^(d)^ Microgels were observed after 7 h. ^(e)^ *r_mc_* value obtained from Equation (6) assuming *f* = 4.

**Table 2:** Results obtained for System B, based on 2,2′-bis(acrylamido)acetic acid (BAC, a_2_), piperazine (P, b_2_), 1,2-diaminoethane (EDA, assumed as b_4_), and morpholine (M, b_1_).

N°	BAC (mmol)	EDA (mmol)	P (mmol)	M (mmol)	Excess Function ^(a)^	*r_c_* ^(b)^	*r* ^(c)^	*r-r_c_*	${\bar{M}}_{n}$ (10^−3^)	${\bar{M}}_{w}$ (10^−3^)
2.1	2.6250	0.3750	3.0000	-	N-H	0.714	0.700	−0.014	4.7	6.1
2.2	2.4975	0.3750	3.0000	-	N-H	0.714	0.667	−0.047	3.9	5.7
2.3	3.1875	0.3750	3.0000	-	N-H	0.714	0.850	+0.136	- ^(d)^	- ^(d)^
2.4	1.6665	0.3750	3.0000	-	N-H	0.714	0.667	−0.047	2.8	4.3
2.5	4.9342	0.3750	3.0000	-	=	0.714	0.760	+0.046	- ^(d)^	- ^(c)^
2.6	5.1370	0.3750	3.0000	-	=	0.714	0.730	+0.016	- ^(d)^	- ^(d)^
2.7	5.6222	0.3750	3.0000	-	=	0.714	0.667	−0.047	2.2	3.5
2.8	3.9280	0.4550	3.0000	2.0000	N-H	0.857 ^(e)^	0.800	−0.057	3.4	4.6
2.9	3.5000	1.3333	3.0000	-	N-H	0.515	0.618	+0.103	3.4	5.3
2.10	3.3333	1.3333	3.0000	-	N-H	0.515	0.588	+0.073	2.9	4.7
2.11	4.2500	1.3333	3.0000	-	N-H	0.515	0.750	+0.235	- ^(d)^	- ^(d)^
2.12	5.0000	0.8889	3.0000	-	=	0.515	0.756	+0.241	- ^(d)^	- ^(d)^
2.13	4.8000	1.3333	3.0000	2.0000	N-H	0.606 ^(e)^	0.720	+0.114	3.1	4.2

^(a)^ N-H: amine function; = acrylamide functions. ^(b)^ Critical compositional ratio obtained from Equation (4) assuming *f* = 4. ^(c)^ Compositional ratio adopted. ^(d)^ Microgels were observed after 7 h. ^(e)^ *r_mc_* values obtained from Equation (6) assuming *f* = 4.

**Table 3:** Results obtained for the System B, based on 2,2′-bis(acrylamido)acetic acid (BAC, a_2_), piperazine (P, b_2_), 1,2-diaminoethane (EDA, assumed as b_3_), and morpholine (M, b_1_).

N°	BAC (mmol)	EDA (mmol)	P (mmol)	M (mmol)	Excess Function ^(^^a)^	*r_c_* ^(b)^	*r* ^(c)^	*r-r_c_*	${\bar{M}}_{n}$ (10^−3^)	${\bar{M}}_{w}$ (10^−3^)
3.1	2.6250	0.3750	3.0000	-	N-H	0.864	0.737	−0.127	4.7	6.1
3.2	2.4975	0.3750	3.0000	-	N-H	0.864	0.701	−0.163	3.9	5.7
3.3	3.1875	0.3750	3.0000	-	N-H	0.864	0.895	+0.031	- ^(d)^	- ^(d)^
3.4	1.6665	0.3750	3.0000	-	N-H	0.864	0.701	−0.163	2.8	4.3
3.5	4.9342	0.3750	3.0000	-	=	0.864	0.736	−0.128	- ^(d)^	- ^(d)^
3.6	5.1370	0.3750	3.0000	-	=	0.864	0.693	−0.161	- ^(d)^	- ^(d)^
3.7	5.6222	0.3750	3.0000	-	=	0.864	0.634	−0.230	2.2	3.5
3.8	3.9280	0.4550	3.0000	2.0000	N-H	1.000 ^(e)^	0.839	−0.161	3.4	4.6
3.9	3.5000	1.3333	3.0000	-	N-H	0.714	0.700	−0.014	3.4	5.3
3.10	3.3333	1.3333	3.0000	-	N-H	0.714	0.667	−0.047	2.9	4.7
3.11	4.2500	1.3333	3.0000	-	N-H	0.714	0.850	+0.136	- ^(d)^	- ^(d)^
3.12	5.0000	0.8889	3.0000	-	=	0.714	0.667	−0.048	-^(d)^	- ^(d)^
3.13	4.8000	1.3333	3.0000	2.0000	N-H	0.857 ^(e)^	0.800	−0.057	3.1	4.2

^(a)^ N-H: amine function; = acrylamide functions. ^(b)^ Critical compositional ratio obtained from Equation (4) assuming *f* = 3. ^(c)^ Compositional ratio adopted. ^(d)^ Microgels were observed after 7 h. ^(e)^ *r_m__c_* values obtained from Equation (6) assuming *f* = 3.

**Table 4:** Hydrodynamic diameter (Z-average), polydispersity (PD), and Zeta-potential (ζ) of microgels by dynamic light scattering measurements.

N°	Z-Average (nm)	PD	ζ (mV)
2.3	481	0.288	+21.1 ± 2.4
2.5	427	0.215	+28.0 ± 3.1
2.6	398	0.187	+18.2 ± 1.3
2.11	473	0.238	+15.9 ± 3.6
2.12	528	0.270	+24.3 ± 1.3
1.3	356	0.211	+21.8 ± 4.9
1.4	294	0.239	+25.1 ± 4.4
1.5	311	0.225	+27.5 ± 3.5

## Data Availability

The data presented in this study are available on request from the corresponding author.
